# Educational spaces for teacher training in nursing at the Unified Health System Vocational Schools

**DOI:** 10.1590/0034-7167-2024-0630

**Published:** 2026-08-07

**Authors:** Juliana Costa Ribeiro-Barbosa, Gilberto Tadeu Reis da Silva, Vânia Marli Schubert Backes, Elaine Kelly Nery Carneiro-Zunino, Daniela Maysa de Souza

**Affiliations:** I Universidade Federal do Recôncavo da Bahia. Santo Antônio de Jesus, Bahia, Brazil; II Universidade Federal da Bahia. Salvador, Bahia, Brazil; III Universidade Federal de Santa Catarina. Florianópolis, Santa Catarina, Brazil; IV Universidade Regional de Blumenau. Blumenau, Santa Catarina, Brazil

**Keywords:** Faculty, Teacher Training, Education, Nursing, Associate, Nursing, Unified Health System., Docente, Formación del Profesorado, Graduación en Auxiliar de Enfermería, Enfermería, Sistema Único de Salud.

## Abstract

**Objectives:**

to identify the pedagogical spaces constituted in the Unified Health System Vocational Schools and their hindering factors for professor training in nursing.

**Methods:**

a descriptive qualitative study, conducted with pedagogical coordinators from five schools located in the North, Northeast, and South regions of Brazil. Data were collected in 2019 through semi-structured interviews and subjected to thematic content analysis using Qualitative Data Analysis Software.

**Results:**

pedagogical spaces are concentrated in three meetings (before, during, and at the end of the teaching activity), aligned with the beginning and end of the subject/module. Temporary teaching staff and the absence of a strengthened network were factors that hindered the implementation of these meetings.

**Final Considerations:**

by promoting training opportunities, schools acknowledge the need for more professional and expert teaching practices. Overcoming the challenges highlighted is crucial for improving teaching performance, leading to enhanced professional training and healthcare.

## RESUMEN

**Objetivos:**

identificar los espacios pedagógicos constituidos en las Escuelas Técnicas del Sistema Único de Salud y sus factores que dificultan la formación docente en enfermería.

**Métodos:**

estudio cualitativo descriptivo, realizado con coordinadores pedagógicos de cinco escuelas ubicadas en las regiones Norte, Nordeste y Sur de Brasil. Los datos se recopilaron en 2019 mediante entrevistas semiestructuradas y se sometieron a análisis de contenido temático mediante un software de análisis de datos cualitativos.

**Resultados:**

los espacios pedagógicos se concentran en tres reuniones (antes, durante y al final de la actividad docente), alineadas con el inicio y el final de la asignatura/módulo. La temporalidad del profesorado y la ausencia de una red de contactos fortalecida fueron factores que dificultaron la implementación de estas reuniones.

**Consideraciones Finales:**

al promover oportunidades de formación, las escuelas reconocen la necesidad de prácticas docentes más profesionales y expertas. Superar los desafíos señalados es crucial para mejorar el desempeño docente, lo que se traduce en una mejor formación profesional y una mejor atención sanitaria.

## INTRODUCTION

Pedagogical practices allow professors, among other possibilities, to critically reflect on their own performance and teaching, as they create spaces for discussion and exchange among peers, providing opportunities to look at oneself and others^(1)^. Therefore, they can encompass a broad set of actions and activities ranging from the planning, implementation, and assessment of learning processes to dynamics that extend beyond these. Based on this perspective, for the purposes of this study, pedagogical practices are understood as formative actions and processes developed in spaces intentionally geared towards professor training, assuming a central role in professional knowledge construction and in educational practice qualification.

These pedagogical spaces are therefore configured as places for dialogue, critique, and self-reflection on educational, pedagogical, and teaching practices, enabling collective transformation and redirection of the senses and meanings of teaching and learning^(1)^. In this way, these environments are understood in this research as strategic and powerful tools for professor training and professional development, functioning as essential resources for professor training and practice, enabling reflection on their actions and paths to improvement, mobilizing knowledge and promoting collective transformation towards a qualified teaching practice.

The teaching profession demands the pursuit and acquisition of a set of fundamental and indispensable knowledge, requiring specific training^(2,3)^. Shulman^(4)^, in his Knowledge Base for Teaching theory, the theoretical framework of this study, presents at least seven categories of knowledge necessary for professors to carry out their work: content knowledge; curriculum knowledge; pedagogical content knowledge; general pedagogical knowledge; knowledge of students; knowledge of educational contexts; and knowledge of educational aims, purposes, and values. Shulman therefore argues in favor of the existence of underlying knowledge for professors’ actions, without which teaching practice becomes vague, uncritical, and superficial. Furthermore, he opposes the understanding that teaching is simply about communicating well, mastering content, and applying the results of studies on its effectiveness. In this context, the conception of pedagogical spaces in the school’s daily life, with a view to professor training, can enable the construction of such knowledge for teaching with higher standards and an expert level.

The need for professor training and its continuity becomes even more pronounced in the context of technical nursing education, considering that most professors in vocational education lack teaching qualifications, in addition to the significant role of technical workers in healthcare^(5-7)^. Professor training has a direct impact on professional training. In the context of the Unified Health System Vocational Schools (In Portuguese, *Escolas Técnicas do Sistema Único de Saúde* - ETSUS), this relationship is even more significant, as professor training and performance will result in the formation of workers who are more sensitive to their role in providing humanized nursing care committed to the Unified Health System (In Portuguese, *Sistema Único de Saúde* - SUS), strengthening and consolidating it as a public health policy.

In this context, attention is drawn to the role of a pedagogical coordinator in planning and developing spaces for promoting professor training. A pedagogical coordinator’s primary responsibility extends to the professor training process in the school’s daily life^(8)^. Therefore, this professional, understanding the significance of the formative attribute of their role and performance, contributes to professors’ reflective exercise on their teaching practice in order to improve their work.

## Study relevance

This article is the product of a thesis^(9)^ deposited in the institutional repository (https://repositorio.ufba.br/handle/ri/37618).

## OBJECTIVES

To identify the pedagogical spaces constituted in ETSUS and their hindering factors for professor training in nursing.

## METHODS

### Ethical aspects

This study was approved by the Research Ethics Committee in September 2019, under Opinion 3,556,307, considering Resolutions 466/2012 and 510/2016 of the Brazilian National Health Council.

Five ETSUS located in the North, Northeast, and South regions of Brazil participated in the research. They received a letter of introduction, the complete project, and a declaration of authorization from the co-participating institution.

As for the six respondents in the research, all signed the Informed Consent Form (ICF). To ensure their anonymity, they were identified by the nominal code “Coord”, referring to coordinator, plus an Arabic numeral randomly assigned.

### Study design

This is a qualitative study with a descriptive typology, extracted from a doctoral thesis entitled “*Formação para a docência em enfermagem nas Escolas Técnicas do SUS: possibilidades em Comunidade de Prática*”, defended and approved in 2021. The study followed COnsolidated criteria for REporting Qualitative research precepts.

### Study setting

The study was conducted in five ETSUS located in Acre, Amazonas, Ceará, Paraná, and Santa Catarina, which had ongoing classes in the nursing technician course, a characteristic considered as an inclusion criterion for the school in the research. Brazil has 41 ETSUS; of these, only the five mentioned had classes in the course in focus in 2019.

It is noteworthy that the approach to the 41 ETSUS occurred in January and February 2019, through telephone contact information provided on the Ministry of Health’s portal, which allowed the identification of schools that had ongoing classes in the technical nursing course. In July of that year, in order to invite them to participate in the study, a cover letter, a declaration of authorization from the co-participating institution, and a copy of the project were sent by email.

### Data source

The pedagogical coordinators of the aforementioned ETSUS participated in the study. The inclusion criterion was being responsible for the nursing technician course. The exclusion criterion comprised being on leave, vacation, or absent from work at the time of data collection.

Initial contact with participants was made via email in September 2019, which was mediated by the school’s management and secretariat, and had the sole purpose of inviting them to participate in the research. Moreover, 100% acceptance was obtained; however, after acceptance, the Acre school’s coordinator was dismissed from his position, and due to refusal by the new coordination, the director and the former coordinator of the nursing technical area agreed to participate in the research, thus making a total of six participants. It is worth mentioning that the suggestion to invite the former coordinator of the technical area was recommended by the director himself, as he believed that he could provide complementary information to his findings.

### Data collection and organization

Data collection took place in October and November 2019 through semi-structured interviews conducted via voice call using a mobile phone dialer. The interviews, scheduled according to participant availability, were audio-recorded without video recording.

Prior to the interviews, an email was sent containing, in addition to the ICF, some clarifications about the purpose of the interview, estimated time, method of conducting it via voice call, need for recording the call, transcript validation, and scheduling according to participants’ availability.

To guide the dialogue, a script divided into three sections was used: 1 - coordinator identification; 2 - coordinator academic background; and 3 - performance in pedagogical coordination. This last section focused on conducting qualifications aimed at professor training, from its conception to its execution, including content, frequency, etc.

All interviews were conducted via recorded voice call, as previously mentioned, since pedagogical coordinators were at their respective schools. The mean duration of each call was 23 minutes, totaling two hours and 18 minutes of data collection.

The interviews were transcribed in a predominantly linear fashion. The interviewer was identified by the term “Rese”, referring to the researcher, and the coordinators were identified by “Coord”, followed by a randomly assigned Arabic numeral, such as “Coord1”. Furthermore, each interview transcript had a cover sheet containing identifying information (interviewer, transcriber, and participant), context (date, start time, and duration), and additional information (transcription outline, page numbers, and explanatory notes). The transcriptions, as well as the verification of reliability and validation, also took place in the months mentioned.

It should be emphasized that the voice recordings and their respective transcripts were stored on an external hard drive for the exclusive use of the lead researcher, with restricted access, ensuring the protection of the collected data.

### Data analysis

For data systematization and analysis, thematic content analysis was adopted according to Bardin’s framework^(10)^. This analysis follows three chronological phases: pre-analysis; material exploration; and results processing and interpretation. As a pre-analytical activity, aimed at the initial organization of the research *corpus*, text skimming of the material was carried out. In material exploration, text coding, cutting, enumeration, and aggregation operations were carried out, enabling a representation of the content. Finally, the results were processed and interpreted, aiming to obtain the manifest and latent content in the discourses for inferences and final understanding. It is worth mentioning that Qualitative Data Analysis Software^®^ was used to support this analysis.

From this process, 53 Registration Units (RUs) emerged, organized based on thematic regularities and articulated around core meanings, which gave rise to four subcategories, later grouped into two analytical categories. The subcategories “Pedagogical meetings prior to the start of teaching practice” (16 RUs/six reference sources), “Pedagogical meetings during teaching practice” (15 RUs/four reference sources), and “Pedagogical meetings at the end of teaching practice” (five RUs/three reference sources) comprise the category “Pedagogical spaces for professor training”, bringing together 36 RUs. The subcategory “Hindering factors” (17 RUs/four reference sources) is, separately, part of the category “Hindering factors for professor training”, whose comprehensive and distinct nature makes its maintenance as a subcategory unnecessary.

## RESULTS

The coordinators were between 32 and 61 years old, with no gender predominance. Their undergraduate education was completed, on average, 24 years ago, varying between nursing, business administration, social work, and pedagogy-the latter mentioned by only two participants. Regarding graduate studies, only one coordinator did not have one, while the others had at least one specialization in the field of education. As for how they obtained their positions, only one was through a public competitive examination, and the others were appointed politically or by invitation.

The following categories and subcategories emerged from participants’ statements and present the pedagogical spaces established in ETSUS for professor training and their hindering factors.

### Pedagogical spaces for professor training

The pedagogical spaces established in ETSUS for professor training are concentrated in three meetings, which take place before the start of teaching practice, during it, and at the end of it, according to the three subcategories below:

### Pedagogical meetings prior to the start of teaching practice

The coordinators mentioned that they hold pedagogical meetings prior to professors’ work in the module or subject. These meetings are intended to align professors on the course itself and on the methodologies adopted at the school, in order to provide them with training to teach the classes.

*When professors join the school, we have a moment with them for alignment, a pedagogical meeting, a training session* [...]. (Coord1)

*Before they go to the classroom, we hold a pedagogical meeting. The first meeting is for alignment, of what and how the course will be, the course process so that they can understand the entire course plan and, along with that, the methodologies that we adopt within the school.* (Coord5)

[...] *we organize the pedagogical meetings and each meeting is specific to each module or subject.* [...] *the next pedagogical meeting will be in November for the professors who will teach nursing classes* [...]. (Coord6)

### Pedagogical meetings during teaching practice

Pedagogical meetings during the teaching work are held in schools by coordinators, considering that many professors hold bachelor’s degrees but lack pedagogical training. Therefore, it is necessary to monitor classes’ methodological progress and, based on any identified need, guide professors on how best to work with specific content.

*In reality, it’s part of my job here* [...] *to monitor lesson plans and methodologies* [...]. (Coord2)

[...] *many professors only have a bachelor’s degree; they don’t have the pedagogical complement; and we have to do all this monitoring. And during classes, if there’s any need, we guide professors, we monitor their activity.* (Coord3)

[...] *we provided pedagogical support to professors regarding methodologies and ideas on how to apply a certain scientific basis* [...]. (Coord4)

### Pedagogical meetings at the end of teaching practice

The study indicated that pedagogical meetings, led by coordinators, take place at the end of course modules or at the end of the school year, with the aim of assessing the teaching-learning process. This assessment is based on professors’ own perceptions of classes’ progress, their performance, and the methodologies used-both those that yielded good results and those that require improvement. In these spaces, professors’ needs regarding the development of their work are also raised and discussed, with the central theme revolving around methodological issues and learning assessment.

*We conduct an assessment at the end of each module, and they point out some needs.* [...] *normally, what we have perceived as a need revolves around issues of methodologies, the assessment process* [...]. (Coord3)

[...] *we also had pedagogical meetings at the end of the year.* (Coord4)

[...] *at the end of each module, we hold a pedagogical meeting.* [...] *the first purpose is evaluative: from the progress of the professors who were in that module, how the class is doing, how the teaching-learning process was, what they saw that worked, what didn’t work, the methodologies that were used that worked best and what didn’t work best, what we can improve.* [...] *and along with that, we also check the demands of our professors* [...]. (Coord5)

It should be noted that participants did not mention specific or standardized instruments for assessment, nor any institutional methods other than meetings.

### Hindering factors for professor training

Regarding professor training implementation, coordinators pointed out the existence of hindering factors, among which the temporary teaching staff was highlighted. Schools do not have permanent professors, since hiring is for a fixed term, leading to high turnover and discontinuity in pedagogical training.

[...] *there is great difficulty in implementing pedagogical training due to the way professors are hired. Our courses are offered through a bidding process. We don’t hire the professor directly; we hire a company that will hire the professor* [...] *it’s complicated in the current hiring method. This is a major obstacle.* (Coord2)

[...] *we don’t have a teaching staff. Our professors are temporary. We hire according to the module offered, so there is no employment relationship. So, this is one of our obstacles due to the difficulty in providing pedagogical training for this professor.* [...] *it often happens that the professor quits the day before the component starts, so this forces us to call in a professor who often doesn’t yet have this pedagogical training.* (Coord3)

[...] *many professors were newcomers. For instance, every year, the professor who started with us found a better job, so they left* [...]. *Most schools have a deficient staff and they are also temporary, and there is a very high turnover in schools. There is no continuity* [...]. (Coord4)

In addition to the provisional teaching staff, one of the coordinators also cited the absence of a strengthened network concept as a hindering factor, in the sense that there is no pedagogical identity among the schools. The adoption of a more uniform direction by schools whose paths are similar is necessary so that they can build this identity in their pedagogical processes and have job security.

*In reality, what’s lacking is strengthening the idea of a network.* [...] *the idea is that schools have a direction, despite their autonomy, but that they closely follow the same path.* [...] *so that we can build a common pedagogical identity that provides job security for all schools*. (Coord2)

## DISCUSSION

The study revealed that the pedagogical spaces established in ETSUS for professor training are concentrated in meetings, organized and facilitated by the pedagogical coordinators, which occur at three distinct times: before the start of professors’ work, during it, and at the end of it.

It is worth highlighting that, in understanding that pedagogical spaces provide opportunities for reflection, dialogue and exchange about professors’ practice, in such a way that they enable knowledge production^(1)^, they go beyond the idea that they merely offer finished instructional packages or are simply a means by which professors are instrumentalized, configuring themselves as spaces where professors learn and improve their practice while interacting with each other.

This practice of pedagogical exchange among professors, promoted in spaces for dialogue and continuous training, is essential to strengthen teaching and foster a renewed and contextualized pedagogical practice, as it enables the construction and reconstruction of daily professional life through reflection on practice^(11)^.

In this way, they overcome the focus brought up in coordinators’ speeches, concentrating them on meetings for training and alignment, for instance. The understanding underlined above conveys the idea not of training and aligning professors in institutional methodologies and rules, but rather of enabling a space for sharing and reflecting on practice, generating transformation/learning and the constitution of pedagogical knowledge for teaching.

It is recognized that initial alignment is necessary to establish common guidelines and ensure the organization of teaching practices. However, it is essential that this alignment goes beyond the mere adaptation of professors to institutional norms, promoting spaces that encourage critical reflection, exchange of knowledge, and adaptation of methodologies to the educational context demands.

It is also important to bring into this discussion the need for investment and institutional support in professor continuing education, creating more training opportunities, considering that teaching is a profession that requires specific training^(3,12)^. Shulman^(4)^, in presenting the Basic Knowledge for Teaching theoretical framework, advocates for the professionalization of teaching, pointing out the categories of knowledge necessary for teaching. Therefore, it is necessary for professors to have this understanding and seek to acquire knowledge that transcends the disciplinary nature of knowledge.

The indispensability of this investment and support is also evident in the fact that most professors working in technical vocational education, including in the context of ETSUS, do not have training aimed at becoming professors^(5,12,13)^. Thus, it becomes evident that it is essential for professors themselves to seek training for their work that goes beyond the specific knowledge of their area, subject, and the schools that employ them, as well as the need for these schools to continuously develop training processes.

Furthermore, the relevance of a pedagogical coordinator’s work in these professor training processes is noteworthy. In their work, this professional plans, creates, and develops actions and strategies that support and enhance professor learning and, consequently, the essential training for the teaching profession^(8)^. In this way, pedagogical coordinators, aware of the responsibility inherent in their work, contribute to a more experienced teaching knowledge/practice.

In the present study, before professors began their teaching activities, their participation in pedagogical meetings was observed, reserved to promote alignment on the course in which they are allocated and on the teaching-learning methodologies that are adopted at the school, in order to provide them with training to teach classes. ETSUS center their teaching-learning process on active methodologies, prioritizing problem-solving and interdisciplinary strategies, directing them towards the local-regional context in which they are inserted^(7,14)^. Therefore, it is essential to develop these preliminary meetings, not only to situate and familiarize them with the school and the course, but also, and above all, to enable the approach and appropriation of active teaching-learning methods, with which they are often unfamiliar.

Active learning methodologies are fundamental for promoting meaningful learning, as they place students as leading actors of their own learning process, encouraging critical thinking, problem-solving, and the collective construction of knowledge. For professors, these methodologies require a reflective and adaptive mediating stance that demands constant review of their practice, adaptation to student needs and the local context, as well as the development of skills to conduct interactive and interdisciplinary dynamics. Thus, the appropriation of these methodologies, fostered in pedagogical meetings, represents a central element for the qualification of teaching performance and for the effectiveness of the teaching-learning process.

It is also important to keep in mind that, in these meetings, professors may have the opportunity to learn about ETSUS and the principles on which their educational processes are based. It is well known that ETSUS occupy a significant place in health education for SUS, as they are committed to basing it on the organizational and doctrinal principles of this system^(7,14)^. Therefore, professors will become aware of and sensitized to this guidance so that they can join efforts to ensure that training processes are planned and developed so that future professionals perform their work in response to the population’s health needs and in accordance with SUS principles, as well as with critical thinking, ethics, and humanization.

Regarding prior pedagogical meetings, many professors working in vocational schools have not been trained for this work, as already mentioned. Their entry into teaching occurs somewhat prematurely and without the specific and necessary training^(13,15)^. Thus, prior pedagogical preparation allows professors to expand their knowledge, leading to the development of a more qualified and expert teaching practice. This directly impacts the teaching process, contributing to meaningful learning.

A study conducted within the context of undergraduate nursing programs indicated that nurses’ training is predominantly focused on patient care, with limited emphasis on preparation for teaching. This contributes, especially at the beginning of their teaching careers, to the adoption of pedagogical practices based on traditional methods^(16)^. This evidence reinforces the need to create educational spaces focused on professor professional development, as well as the institutional offering of courses that promote the incorporation of pedagogical and technological resources into teaching practice, in order to foster critical and reflective learning by students.

In this context, the nursing degree plays a fundamental role, seeking to train professors for vocational technical education at the secondary level, articulating content that encompasses both training the generalist nurse and the nurse educator^(17)^. However, its availability is quite limited compared to the bachelor’s degree. A survey conducted in August 2019 indicated that the teaching degree corresponds to only 1% (12) of the 1,168 undergraduate nursing courses in Brazil^(18)^. This reality highlights the continued presence of bachelor’s degree holders in teaching, which reinforces the fragility of recognizing teaching as a profession that demands specific training, due to its unique characteristics and complexity.

In this context of professor training, ETSUS also develop pedagogical meetings throughout a professor’s career, with the purpose of monitoring and verifying the progress of classes and guiding the professor on how best to work with specific content, according to their needs and concerns. This pedagogical support allows professors to reflect on their own practice, which promotes learning and maturity, thus constituting a strategy for strengthening and improving teaching performance^(15)^. From this perspective, it is possible for professors to seek new knowledge and teaching-learning strategies, improving them with creativity and critical thinking, and reorienting their educational practices.

It is opportune to emphasize, once again, how indispensable and pressing it is, for the exercise of teaching, to seek knowledge that exceeds the limits of the depth of understanding of a particular subject. By pointing out the seven categories of Basic Knowledge for Teaching, Shulman^(4)^ highlights knowledge that is fundamental to the professor’s own understanding, i.e., to understanding what should be learned and how it should be taught. This understanding is a key element for student learning. In other words, professor training and performance have repercussions on students’ education.

Specifically when it comes to technical training, the pursuit of knowledge that transcends nursing/health is fundamental for nursing faculty to have a more qualified performance and contribute to professional training improvement and nursing strengthening^(19)^. Furthermore, it is also essential, due to the significant number and relevance of the duties of technical workers in healthcare^(7,20)^. Therefore, education, especially nursing education, needs to be approached with the responsibility and complexity that the profession demands, as it has specificities that require training for its practice.

Finally, and still within this context of professor training, ETSUS hold pedagogical meetings at the end of professors’ performance, after the completion of the course modules or academic year, to assess their work. The possibility of reflecting on their actions allows professors to analyze themselves and their professional practice from a (trans)formation perspective^(8)^. Therefore, the continuous professional development process for professors is related to feedback on their performance as professors, constituting powerful instruments for self-reflection and the pursuit of knowledge and qualification towards a more experienced teaching practice.

In these meetings, professors also have the opportunity to share demands from their practice, which are mostly directed towards teaching and assessment methodologies. It becomes evident that, for teaching, knowledge that goes beyond content is necessary. In support, Shulman^(4)^ states that general pedagogical knowledge is one of the constitutive knowledge bases for teaching. This knowledge allows professors to more easily access and more appropriately choose teaching methodologies, as well as to appropriately select instruments for learning assessment^(2)^. Therefore, professors are required to have continuous and ongoing training that allows them to achieve the necessary qualifications for teaching.

Concerning the development of pedagogical meetings, the coordinators mentioned some hindering factors, such as the temporary teaching staff and the absence of a strengthened network concept.

As for the temporary teaching staff, given that the hiring is for a fixed term, the difficulty lies in the discontinuity of professors’ pedagogical training. Professor affiliation with vocational schools commonly occurs temporarily, which leads to a high turnover of professors^(12)^. Certainly, such rotation compromises both the continuity of teaching and the improvement of training processes.

A study conducted in Rio Grande do Sul found that the lack of secure employment in the careers of vocational education professors is a reality, with approximately 64% to 89.4% of professors holding more than one position, negatively impacting pedagogical practice, which is a complex activity that demands daily preparation time, exceeding professors’ workload^(21)^.

The coordinators, when pointing out this difficulty related to the type of professor contracts, draw attention to the need to review the types of contractual relationships established by vocational education schools. Such a review is essential given the instability generated by temporary contracts, which hinder the retention and continuous development of professors in the educational environment. Added to this is the precariousness of these contractual relationships, frequently marked by low salaries and the absence of a structured career plan. This reality compromises not only professors’ engagement and appreciation, but also education quality, since it prevents the building of lasting bonds with the institution and with students.

In reference to the absence of a strengthened network concept, this hindering factor, in the coordinator’s view, leads to a lack of a common pedagogical identity among ETSUS and insecurity in their work processes. Therefore, it is pointed out that there is a need for schools to follow similar paths, based on a more uniform direction in the conduct of their pedagogical processes.

It is known that ETSUS are organized in a network, the SUS Vocational Schools Network (In Portuguese, *Rede de Escolas Técnicas do SUS* - RET-SUS), established in the 2000s, whose legal framework was updated in 2009 under Ordinance 2,970. Currently, the network consists of 41 schools, present in all Brazilian states. Among other objectives, it aims to integrate the ETSUS through the sharing of knowledge and information of common interest to schools^(22)^. From this perspective, it is evident that RET-SUS facilitates a sense of unity and integration among schools, regardless of their geographical distance. This results in a state of alignment among them.

However, given the aforementioned difficulty, the sense of unity and positioning, as well as the integration provided by the network, need to be strengthened so that schools can reflect a convergence in their educational processes, especially those related to professor training. This will result in a similar and convergent pedagogical profile, which will provide security to managers and coordinators in conducting their work.

### Study limitations

It is worth mentioning that the study limitations relate to the data collection being carried out exclusively with the coordinators, the lack of verification of lesson plans in relation to the teaching methodology proposed by ETSUS, as well as the absence of analysis of assessments carried out by professors and students. Therefore, it is recommended that future research be conducted with the aim of broadening the understanding of the object/theme, incorporating different perspectives and data sources, in order to strengthen the validity and scope of results.

### Contributions to nursing

This study highlights the need for recognition and improvement of teaching professionalization, pointing especially to the importance and indispensability of professor training for teaching in technical nursing education, particularly in the context of ETSUS. Teaching’s responsibility, complexity, and challenges make professor training a compelling and continuous demand, considering professional training quality and nursing care.

## FINAL CONSIDERATIONS

The pedagogical spaces established in for professor training focused on pedagogical meetings that took place before, during, and at the end of teaching practice. Factors hindering the development of this training included a temporary teaching staff and the absence of a strengthened network concept.

Qualification for teaching is a real need, given that teaching is a profession requiring specific knowledge and training. In the context of technical nursing education, this need becomes even more evident, as the professor is directly involved in the training of this worker, who is essential for care within the context of SUS.

ETSUS, through its pedagogical coordinators, when planning and implementing pedagogical meetings aimed at promoting professor qualification, acknowledges the necessity and importance of developing strategies in daily school life that enable the exchange of knowledge among professors so that they can perform their work in a more professional and expert manner. It is worth highlighting how essential professors’ involvement and commitment to their own professional development is, whether through participation in institutional training or the personal pursuit of improvement.

It is evident that challenges do exist, and overcoming them is a decisive element in training qualified professors who contribute to training future professionals committed to providing healthcare within and for SUS. Therefore, the recommendation is to think about and implement more convergent actions that are interconnected and networked, aiming at formative possibilities and addressing the challenges that are currently presented.

## Data Availability

The research data are available within the article.

## References

[1] Franco MARS. (2016). Prática pedagógica e docência: um olhar a partir da epistemologia do conceito. Rev Bras Estud Pedagógicos.

[2] Backes VMS, Menegaz JC, Miranda FAC, Santos LMC, Cunha AP, Patrício SS. (2017). Lee Shulman: contributions to research on teacher training. Texto Contexto Enferm.

[3] Nóvoa A. (2017). Firmar a posição como professor, afirmar a profissão docente. Cad Pesqui.

[4] Shulman LS. (2014). Conhecimento e ensino: fundamentos para a nova reforma. Cad Cenpec.

[5] Souza DM, Backes VMS, Lazzari DD, Santos LMC, Martini JG. (2020). Conhecimento Pedagógico de Conteúdo de docentes de enfermagem novatos na educação técnica de nível médio. Rev Bras Enferm.

[6] Corrêa AK, Sordi MRL. (2018). The secondary technical-professional education in the unified health system and the teacher training policy. Texto Contexto Enferm.

[7] Ribeiro-Barbosa JC, Silva GTR, Amestoy SC, Silva CCR, Silva RMO, Backes VMS (2020). Escolas técnicas do Sistema Único de Saúde: uma análise da formação em enfermagem. Rev Esc Enferm USP.

[8] Campos PRI, Aragão AMF. (2016). A coordenadora pedagógica e a formação docente: possíveis estratégias de atuação. Rev Educ PUC-Campinas.

[9] Ribeiro-Barbosa JC. (2021). Formação para a docência em enfermagem nas escolas técnicas do SUS: possibilidades em comunidade de prática.

[10] Bardin L. (2016). Análise de conteúdo.

[11] Medeiros RO, Marin MJS, Lazarini CA, Castro RM, Higa EFR. (2022). Formação docente em metodologias de aprendizagem ativa. Interface (Botucatu).

[12] Sgarbi AKG, Missio L, Renovato RD, Hortelan MPSM. (2018). Enfermeiro docente no ensino técnico de enfermagem. Laplage Rev.

[13] Siqueira MCG, Leopardi MT. (2016). O processo ensino-aprendizagem na formação de trabalhadores do sus: reflexões a partir da experiência da ETSUS. Trab Educ Saúde.

[14] Tafner DPOV, Reibnitz KS, Lazzari DD. (2016). Implementation of the integrality principle in technical nursing courses at schools in the SUS network. Texto Contexto Enferm.

[15] Souza DM, Backes VMS, Prado ML. (2016). Formação docente na educação profissional técnica de nível médio: uma revisão integrativa da literatura. Interfaces da Educ.

[16] Ribeiro JF, Costa JML, Silva MAC, Luz VLES, Veloso MV, Ribeiro ALI (2018). Prática pedagógica do enfermeiro na docência do ensino superior. Rev Enferm UFPE.

[17] Ribeiro-Barbosa JC, Silva GTR, Backes VMS, Vieira SL, Chaves MCRF, Paiva JMM. (2022). Training-professional profile of nurses teaching at the Technical Schools of the Unified Health System. Rev Bras Enferm.

[18] Rampellotti LF, Pasqualli R. (2020). O bom professor enfermeiro: o olhar dos estudantes de cursos técnicos acerca da prática docente. Rev Exitus.

[19] Souza DM, Backes VMS, Lazzari DD, Martini JG. (2018). Pedagogical preparation of nursing professors for professional secondary technical education. Rev Bras Enferm.

[20] Conselho Federal de Enfermagem (COFEn) (2025). Enfermagem em Números.

[21] Franco MT, Fernandes MTC, Millão LF. (2020). Perfil de enfermeiros-professores da educação profissional técnica de nível médio em enfermagem. Saúde Coletiva.

[22] Leite ICM, Mourão LC, Almeida ACV. (2020). Teaching implications in the pedagogical training of a technical school. Rev Bras Enferm.

