# Supplementary material for: Educational spaces for teacher training in nursing at the Unified Health System Vocational Schools
Source: Rev Bras Enferm. 2026 Aug 7;79:e20240630. doi: 10.1590/0034-7167-2024-0630 (PMC13450831; doi:10.1590/0034-7167-2024-0630)
Supplement: Translation

# Translation: Espaços pedagógicos de formação docente em enfermagem nas Escolas Técnicas do Sistema Único de Saúde

**DOI:** 10.1590/0034-7167-2024-0630pt

**Authors:** Juliana Costa Ribeiro-Barbosa, Gilberto Tadeu Reis da Silva, Vânia Marli Schubert Backes, Elaine Kelly Nery Carneiro-Zunino, Daniela Maysa de Souza

**Affiliations:** I Universidade Federal do Recôncavo da Bahia. Santo Antônio de Jesus, Bahia, Brasil; II Universidade Federal da Bahia. Salvador, Bahia, Brasil; III Universidade Federal de Santa Catarina. Florianópolis, Santa Catarina, Brasil; IV Universidade Regional de Blumenau. Blumenau, Santa Catarina, Brasil

**Keywords:** Docente, Capacitação de Professores, Educação Técnica em Enfermagem, Enfermagem, Sistema Único de Saúde.

## Abstract

**Objetivos:**

identificar os espaços pedagógicos constituídos nas Escolas Técnicas do Sistema Único de Saúde e seus fatores dificultadores para formação docente em enfermagem.

**Métodos:**

estudo qualitativo descritivo, realizado com coordenadores pedagógicos de cinco escolas localizadas nas regiões Norte, Nordeste e Sul do Brasil. Os dados foram coletados em 2019, por entrevista semiestruturada, e submetidos à análise de conteúdo temática com suporte do *Qualitative Data Analysis Software*.

**Resultados:**

os espaços pedagógicos concentram-se em três encontros (antes, durante e ao final da atuação docente), alinhados ao início e término da disciplina/módulo. O quadro provisório de professores e a ausência de uma rede fortalecida foram fatores que dificultaram a implementação destes encontros.

**Considerações Finais:**

as escolas, ao promoverem espaços formativos, assumem a necessidade de um exercício docente mais profissional e experto. Superar os desafios evidenciados é decisivo para qualificar a atuação docente, com consequente melhoria da formação profissional e cuidado em saúde.

## INTRODUÇÃO

As práticas pedagógicas permitem ao professor, entre outras possiblidades, a reflexão crítica sobre sua própria atuação e seu próprio ensino, na medida em que criam espaços de discussão e troca entre pares, oportunizando o olhar sobre si e sobre o outro^(1)^. Sendo assim, podem abranger um conjunto amplo de ações e atividades que vão desde o planejamento, operacionalização e avaliação dos processos de aprendizagem até dinâmicas que se situam para além destes. Com base nessa perspectiva, para os fins deste estudo, as práticas pedagógicas são, portanto, compreendidas como ações e processos formativos desenvolvidos em espaços intencionalmente voltados à formação docente, assumindo um papel central na construção de saberes profissionais e na qualificação da prática educativa.

Esses espaços pedagógicos se configuram, por conseguinte, como lugares de diálogo, crítica e autorreflexão das práticas educativas, pedagógicas e docentes, possibilitando transformação coletiva e redirecionamento dos sentidos e significados do ensino e da aprendizagem^(1)^. Dessa maneira, são entendidos, nesta pesquisa, como ambientes estratégicos e potentes de formação e desenvolvimento profissional dos professores, funcionando como subsídios essenciais à formação e atuação na docência, ao viabilizarem reflexões sobre suas ações e caminhos para o aprimoramento, mobilizando saberes e promovendo a transformação coletiva para um exercício docente qualificado.

A profissão de professor reivindica a busca e aquisição de um agregado de conhecimentos que são estruturantes e indispensáveis, requisitando uma formação específica^(2,3)^. Em sua teoria do Conhecimento Base para o Ensino, referencial teórico deste estudo, Shulman^(4)^ apresenta pelo menos sete categorias de conhecimentos necessários ao professor para o exercício do seu trabalho: conhecimento do conteúdo; conhecimento curricular; conhecimento pedagógico do conteúdo; conhecimento pedagógico geral; conhecimento dos alunos; conhecimento dos contextos; e conhecimento dos objetivos e valores educacionais. Advoga, portanto, em favor da existência de conhecimentos subjacentes à atuação do professor, sem os quais a prática docente se torna vaga, acrítica e superficial. Para mais, contrapõe-se a compreensão de que o ensino é comunicar-se bem, dominar um conteúdo e aplicar resultados de estudos sobre sua eficácia. Diante disto, a concepção de espaços pedagógicos no dia a dia da escola, tendo em vista a formação de professores, pode viabilizar a construção de tais conhecimentos para um ensino com padrões mais elevados e de nível experto.

A necessidade da formação para a docência e sua continuidade se torna ainda mais acentuada no contexto da educação técnica em enfermagem ao se considerar que a maioria dos professores que lecionam na educação profissional não possui qualificação para o magistério, além do expressivo espaço ocupado pelo trabalhador técnico no cuidado em saúde^(5-7)^. A formação do professor reverbera na formação dos profissionais. No cenário das Escolas Técnicas do Sistema Único de Saúde (ETSUS), essa relação alcança maior magnitude, porquanto a formação e atuação docente irão repercutir na formação de trabalhadores mais sensíveis do seu papel para uma assistência em enfermagem humanizada e comprometida com o Sistema Único de Saúde (SUS), fortalecendo-o e consolidando-o como política pública de saúde.

Nesse contexto, chama-se atenção para a atuação da coordenação pedagógica no planejamento e desenvolvimento de espaços para promoção da formação docente. A responsabilidade primordial do coordenador pedagógico perpassa o processo formativo do professor no cotidiano da escola^(8)^. Portanto, este profissional, na compreensão da significância do atributo formativo de sua função e atuação, contribui com o exercício reflexivo do professor sobre sua prática docente no sentido de aprimorar seu trabalho.

## Relevância do estudo

Este artigo é produto de tese^(9)^ depositada no repositório institucional (https://repositorio.ufba.br/handle/ri/37618).

## OBJETIVOS

Identificar os espaços pedagógicos constituídos nas ETSUS e seus fatores dificultadores para formação docente em enfermagem.

## MÉTODOS

### Aspectos éticos

Este estudo foi aprovado pelo Comitê de Ética em Pesquisa em setembro de 2019, sob Parecer nº 3.556.307, considerando as Resoluções nº 466/2012 e nº 510/2016 do Conselho Nacional de Saúde.

Participaram da pesquisa cinco ETSUS situadas nas regiões Norte, Nordeste e Sul do Brasil, as quais receberam carta de apresentação, projeto na íntegra e declaração de autorização da instituição coparticipante.

Quanto aos seis respondentes da pesquisa, todos assinaram o Termo de Consentimento Livre e Esclarecido (TCLE). Para assegurar o seu anonimato, foram identificados pelo código nominal “Coord”, referente a coordenador(a), acrescido de um algarismo arábico disposto aleatoriamente.

### Tipo de estudo

Estudo com abordagem qualitativa e tipologia descritiva extraído de tese de doutorado intitulada “Formação para a docência em enfermagem nas Escolas Técnicas do SUS: possibilidades em Comunidade de Prática”, defendida e aprovada em 2021. O estudo considerou os preceitos do *COnsolidated criteria for REporting Qualitative research*.

### Cenário do estudo

O estudo teve como cenário cinco ETSUS localizadas no Acre, Amazonas, Ceará, Paraná e Santa Catarina, que possuíam turmas em andamento do curso técnico de enfermagem, característica considerada como critério de inclusão da escola na pesquisa. O Brasil tem 41 ETSUS; destas, apenas as cinco citadas possuíam turmas do curso em foco em 2019.

Salienta-se que a aproximação das 41 ETSUS ocorreu nos meses de janeiro e fevereiro de 2019, por meio de contato telefônico informado no portal no Ministério da Saúde, o que permitiu a identificação das escolas que possuíam turma em andamento do curso técnico de enfermagem. Em julho do referido ano, no intuito de convidá-las a participar do estudo, foram enviadas por e-mail carta de apresentação, declaração de autorização da instituição coparticipante e cópia do projeto.

### Fonte de dados

Participaram do estudo os coordenadores pedagógicos das ETSUS mencionadas. Constituiu-se como critério de inclusão responder pelo curso técnico de enfermagem. Constituiu-se como critério de exclusão estar de licença, férias ou afastado do trabalho no momento da coleta de dados.

O contato inicial com os participantes foi feito através de *e-mail* em setembro de 2019, o qual foi intermediado pela diretoria e secretaria da escola, e teve, exclusivamente, o intuito de convidá-los a participar da pesquisa. Obteve-se 100% de aceitação, contudo, após o aceite, o coordenador da escola do Acre foi desligado de sua função e, por recusa da nova coordenação, o diretor e antigo coordenador da área técnica de enfermagem aceitaram participar da pesquisa, perfazendo, assim, um total de seis participantes. É válido mencionar que a indicação para realização do convite ao antigo coordenador da área técnica foi recomendada pelo próprio diretor, por entender que ele poderia fornecer informações complementares às suas.

### Coleta e organização dos dados

A coleta de dados aconteceu nos meses de outubro e novembro de 2019, por meio de entrevista semiestruturada, através de chamada de voz, realizada diretamente pelo discador do telefone móvel. As entrevistas, agendadas conforme disponibilidade do participante, foram gravadas em áudio, sem a utilização de recurso de vídeo.

Anterior à realização das entrevistas, foi enviado *e-mail* contendo, além do TCLE, alguns esclarecimentos sobre o objetivo da entrevista, tempo estimado, meio de realização por chamada de voz, necessidade de gravação da chamada, validação da transcrição, e agendamento conforme disponibilidade de data e horário do participante.

Para nortear o diálogo, utilizou-se um roteiro dividido em três seções: 1 - identificação do coordenador; 2 - formação acadêmica do coordenador; e 3 - atuação na coordenação pedagógica. Esta última enfocou a realização de qualificações direcionadas à formação docente, desde sua idealização até a execução, passando pelos conteúdos, periodicidade, etc.

Todas as entrevistas foram realizadas por chamada de voz gravada, como referido anteriormente, uma vez que os coordenadores pedagógicos se encontravam em suas respectivas escolas, com tempo médio de duração de 23 minutos, perfazendo duas horas e 18 minutos de coleta de dados.

As entrevistas foram transcritas de forma predominantemente linear, sendo que, para identificação da entrevistadora, foi utilizado o termo “Pesq”, alusivo à pesquisadora, e para os coordenadores, empregou-se “Coord”, seguido de um algarismo arábico posto de maneira aleatória, como “Coord1”. Ademais, cada transcrição de entrevista possuía uma folha de rosto na qual constavam informações de identificação (entrevistadora, transcritora e participante), do contexto (data, horário de início e tempo de duração) e adicionais (esquema de transcrição, número de páginas e notas explicativas). As transcrições, assim como a conferência de fidedignidade e validação, também ocorreram nos meses citados.

Sublinha-se que as gravações de voz e suas respectivas transcrições foram armazenadas em um *hard disk* externo de uso exclusivo da pesquisadora responsável, com acesso restrito, garantindo a proteção dos dados coletados.

### Análise dos dados

Para sistematização e análise dos dados, adotou-se a análise de conteúdo temática conforme referencial de Bardin^(10)^. Tal análise segue três pólios cronológicos: pré-análise; exploração do material; e tratamento dos resultados e interpretação. Como atividade pré-analítica, destinada à organização inicial do *corpus* da pesquisa, realizou-se a leitura flutuante do material. Referente à exploração do material, realizaram-se as operações de codificação de recorte, enumeração e agregação do texto, possibilitando uma representação do conteúdo. Por fim, seguiu-se para o tratamento dos resultados e interpretação, tendo em vista a obtenção dos conteúdos manifestos e latentes nos discursos para as inferências e compreensão final. É oportuno dizer que o *software* de análise de dados qualitativos *Qualitative Data Analysis Software* ^®^ foi utilizado como suporte para esta análise.

Desse processo, emergiram 53 Unidades de Registro (URs), organizadas com base em regularidades temáticas e articuladas em torno de núcleos de sentido, que originaram quatro subcategorias, posteriormente agrupadas em duas categorias analíticas. As subcategorias “Encontros pedagógicos prévios ao início da atuação docente” (16 URs/seis fontes referenciais), “Encontros pedagógicos no decorrer da atuação docente” (15 URs/quatro fontes referenciais) e “Encontros pedagógicos ao final da atuação docente” (cinco URs/três fontes referenciais) compõem a categoria “Espaços pedagógicos para formação docente”, reunindo 36 URs. Já a subcategoria “Fatores dificultadores” (17 URs/quatro fontes referenciais) integra, isoladamente, a categoria “Fatores dificultadores para formação docente”, cujo caráter abrangente e distinto torna desnecessária sua manutenção como subcategoria.

## RESULTADOS

Os coordenadores tinham idade entre 32 e 61 anos, sem predominância entre os sexos. A formação em nível de graduação foi concluída, em média, há 24 anos, variando entre enfermagem, administração de empresas, serviço social e pedagogia - esta última mencionada por apenas dois participantes. Referente à pós-graduação, somente um coordenador não possuía, e os demais tinham pelo menos uma especialização na área da educação. Quanto à ocupação do cargo, somente um foi através de concurso público, e os outros, por indicação política ou convite.

As categorias e subcategorias, a seguir, emergiram das falas dos participantes e apresentam os espaços pedagógicos constituídos nas ETSUS para formação docente e seus fatores dificultadores.

### Espaços pedagógicos para formação docente

Os espaços pedagógicos constituídos nas ETSUS para formação docente se concentram em três encontros, que acontecem, de forma prévia ao início da atuação docente, no decorrer e ao final da mesma, conforme as três subcategorias a seguir:

### Encontros pedagógicos prévios ao início da atuação docente

Os coordenadores referiram que promovem encontros pedagógicos previamente à atuação do professor no módulo ou disciplina. Tais encontros se destinam a um alinhamento sobre o próprio curso e a respeito das metodologias que são adotadas na escola, de modo a proporcionar-lhes um treinamento para ministrar as aulas.

*Quando os professores ingressam na escola, temos um momento com eles para alinhamento, um encontro pedagógico, um momento de treinamento* [...]. (Coord1)

*Antecedendo a eles irem para a sala de aula, nós realizamos um encontro pedagógico. O primeiro encontro é de alinhamento, do que e de como vai ser o curso, o processo do curso, para que ele possa ter entendimento de todo o plano de curso e junto a isso, das metodologias que nós adotamos dentro da escola*. (Coord5)

[...] *nós vamos organizando os encontros pedagógicos e cada encontro específico para cada módulo ou disciplina*. [...] *o próximo encontro pedagógico vai ser em novembro para os docentes que irão ministrar aulas em enfermagem* [...]. (Coord6)

### Encontros pedagógicos no decorrer da atuação docente

Encontros pedagógicos no decorrer do trabalho docente são realizados nas escolas pelos coordenadores, tendo em vista que muitos professores são bacharéis e não têm uma formação pedagógica. Assim, faz-se necessário acompanhar o andamento metodológico das aulas e, a partir de alguma necessidade manifesta, orientar o docente em como melhor trabalhar determinado conteúdo.

*Na realidade, faz parte do rol das minhas funções aqui* [...] *acompanhamento do plano de aula e das metodologias* [...]. (Coord2)

[...] *muitos professores têm só bacharelado; eles não têm a complementação pedagógica; e temos que fazer todo esse acompanhamento. E durante as aulas, alguma necessidade, a gente orienta o professor, acompanha a atividade dele.* (Coord3)

[...] *a gente dava um acompanhamento pedagógico para os professores em relação as metodologias e ideias de como aplicar determinada base científica* [...]. (Coord4)

### Encontros pedagógicos ao final da atuação docente

O estudo apontou que encontros pedagógicos, conduzidos pelos coordenadores, ocorrem ao final dos módulos do curso ou ao término do ano letivo, com o objetivo de avaliar o processo de ensino-aprendizagem. Essa avaliação é construída com base nas percepções dos próprios docentes sobre o andamento das turmas, sua atuação e as metodologias utilizadas - tanto as que apresentaram bons resultados quanto as que demandam aprimoramento. Nesses espaços, também são levantadas e discutidas demandas dos docentes em relação ao desenvolvimento de seu trabalho, cuja temática central gira em torno das questões metodológicas e de avaliação da aprendizagem.

*Nós fazemos uma avaliação ao final de cada módulo, e eles apontam algumas necessidades*. [...] *normalmente, o que a gente tem percebido de necessidade volta-se para as questões das metodologias, do processo de avaliação* [...]. (Coord3)

[...] *tínhamos os encontros pedagógicos também no final do ano*. (Coord4)

[...] *a cada final de módulo, nós realizamos um encontro pedagógico*. [...] *o primeiro intuito é o avaliativo: desde o andamento dos professores que estiveram naquele módulo, como está a turma, como foi o processo de ensino-aprendizagem, o que eles viram que funcionou, que não funcionou, as metodologias que foram utilizadas que mais deu certo e que não deu certo, o que nós podemos aprimorar.* [...] *e junto a isso, a gente também verifica a demanda dos nossos professores* [...]. (Coord5)

Ressalta-se que os participantes não mencionaram instrumentos específicos ou padronizados para avaliação, nem outras formas institucionais além dos encontros.

### Fatores dificultadores para formação docente

Para implementação da formação docente, os coordenadores assinalaram a existência de fatores dificultadores, entre os quais foi apontado o quadro docente provisório. As escolas não dispõem de professores permanentes, porquanto a contratação é por tempo determinado, acarretando enorme rotatividade e descontinuidade da formação pedagógica.

[...] *grande dificuldade de implementar a formação pedagógica pela forma de contratação dos docentes. Nossos cursos se dão por pregão. Não contratamos diretamente o docente; contratamos uma empresa que vai contratar o docente* [...] *é complicado na forma atual de contratação. Esse é um grande dificultador*. (Coord2)

[...] *não temos um quadro de professores. Nossos professores são temporários. Nós contratamos conforme o módulo que é oferecido, então não há vínculo empregatício. Então, esse é um dos nossos entraves por conta da dificuldade em dar uma formação pedagógica pra esse professor*. [...] *acontece, muitas vezes, do professor desistir um dia antes de iniciar o componente, então isso nos obriga a chamar um professor que, muitas vezes, não tem ainda essa formação pedagógica*. (Coord3)

[...] *muitos professores eram novatos. Um exemplo, a cada ano, o professor que começou com a gente encontrava um trabalho melhor, então ele saía* [...]. *A maioria das escolas tem um quadro deficitário e eles são também provisórios, e há uma rotatividade muito grande nas escolas. Não há continuidade* [...]. (Coord4)

Além do quadro provisório de docentes, um dos coordenadores também citou a ausência de uma ideia de rede fortalecida como fator dificultador, no sentido de não haver uma identidade pedagógica entre as escolas. A adoção de um direcionamento mais uniforme pelas escolas, cujos caminhos percorridos estejam próximos, faz-se necessária para que possam construir esta identidade em seus processos pedagógicos e tenham segurança de trabalho.

*Na realidade, está faltando fortalecer a ideia de rede*. [...] *a ideia é que as escolas tenham um direcionamento, apesar de seu espaço de autonomia, mas que trilhem muito próximo o mesmo caminho*. [...] *para que possamos construir uma identidade pedagógica comum e que dê segurança de trabalho a todas as escolas*. (Coord2)

## DISCUSSÃO

O estudo evidenciou que os espaços pedagógicos constituídos nas ETSUS para formação docente se concentram em encontros, organizados e dinamizados pelos coordenadores pedagógicos, que ocorrem em três momentos distintos, acontecendo previamente ao início da atuação do docente, no decorrer e ao final da mesma.

Vale ressaltar que, na compreensão de que os espaços pedagógicos oportunizam reflexão, interlocução e troca sobre a prática do professor, de tal forma que viabilizam a produção de conhecimento^(1)^, extrapolam a ideia de que oferecem meramente pacotes instrucionais acabados ou são simplesmente um meio pelo qual o professor é instrumentalizado, configurando-se como espaços onde os professores aprendem e melhoram sua prática enquanto interagem entre si.

Essa prática de troca pedagógica entre professores, promovida em espaços de diálogo e capacitação contínua, é essencial para fortalecer a docência e favorecer uma prática pedagógica renovada e contextualizada, pois possibilita a construção e reconstrução do cotidiano profissional por meio da reflexão sobre a prática^(11)^.

Dessa maneira, superam o foco trazido nas falas dos coordenadores, concentrando-os em encontros para treinamento e alinhamento, por exemplo. A compreensão sublinhada acima traz a ideia não de treinar e alinhar o professor nas metodologias e regras institucionais, mas sim de viabilizar um lugar de partilha para e na reflexão da prática, gerando transformação/aprendizado e constituição do saber pedagógico para a docência.

Reconhece-se que o alinhamento inicial é necessário para estabelecer diretrizes comuns e garantir a organização das práticas docentes. Contudo, é fundamental que esse alinhamento supere a mera adequação do professor às normas institucionais, promovendo espaços que incentivem a reflexão crítica, troca de saberes e adaptação das metodologias às demandas do contexto educacional.

Cabe também trazer nesta discussão a necessidade de investimento e fomento institucional na formação contínua do professor, constituindo mais espaços formativos, ao considerar que ensino é profissão que requer formação específica^(3,12)^. Shulman^(4)^, ao apresentar o referencial teórico do Conhecimento Base para o Ensino, milita nesta defesa da profissionalização do Ensino, apontando as categorias de conhecimento necessários para ensinar. Portanto, é necessário que o professor tenha esta compreensão e busque adquirir saberes que transcendam a disciplinaridade do saber.

A indispensabilidade deste investimento e fomento também se manifesta ao resgatar que a maioria dos docentes que atuam na educação profissional técnica, inclusive no contexto das ETSUS, não possui formação que vise ao magistério^(5,12,13)^. Assim, fica em evidência a essencialidade dos próprios professores buscarem formação para o exercício de seu trabalho que ultrapasse os conhecimentos específicos da sua área, matéria e das escolas que os contratam, bem como a necessidade destas desenvolverem continuamente processos formativos.

Ademais, notabiliza-se a relevância do trabalho do coordenador pedagógico nesses processos de formação dos professores. Na prática de sua atividade laboral, esse profissional planeja, cria e desenvolve ações e estratégias que subsidiam e potencializam a aprendizagem docente e, consequentemente, a formação indispensável para a profissão de professor^(8)^. Dessa maneira, o coordenador pedagógico, ciente da responsabilidade que reside em seu trabalho, contribui para um saber/fazer docente mais experimentado.

No presente estudo, antes de os professores iniciarem suas atividades de ensino, constatou-se a sua participação em encontros pedagógicos reservados para promover um alinhamento sobre o curso no qual estão alocados e sobre as metodologias de ensino-aprendizagem que são adotadas na escola, de maneira a proporcionar-lhes um treinamento para ministrar aulas. As ETSUS centram seu processo de ensino-aprendizagem nas metodologias ativas, priorizando as estratégias problematizadoras e interdisciplinares, voltando-as para o contexto loco-regional no qual estão inseridas^(7,14)^. Dessa forma, é essencial o desenvolvimento desses encontros prévios, não somente para situá-los e ambientá-los em relação à escola e ao curso, mas também, sobretudo, para viabilizar a aproximação e apropriação de métodos ativos para ensinar-aprender, com os quais, muitas vezes, não estão familiarizados.

As metodologias ativas são fundamentais para promover um aprendizado significativo, pois colocam o aluno como protagonista do seu processo formativo, estimulando o pensamento crítico, a resolução de problemas e a construção coletiva do conhecimento. Para o professor, essas metodologias exigem uma postura reflexiva e adaptativa de mediador que demanda constante revisão da sua prática, adequação às demandas dos estudantes e ao contexto local, além do desenvolvimento de habilidades para conduzir dinâmicas interativas e interdisciplinares. Assim, a apropriação dessas metodologias, fomentada nos encontros pedagógicos, representa um elemento central para a qualificação da atuação docente e para a efetividade do processo de ensino-aprendizagem.

Não se pode perder de vista também o fato de que os professores, nesses encontros, podem ter a oportunidade de conhecer um pouco sobre as ETSUS e em quais fundamentos alicerçam seus processos educativos. É sabido que as ETSUS ocupam lugar expressivo na formação em saúde para o SUS, por comprometer-se em baseá-la nos princípios organizativos e doutrinários desse sistema^(7,14)^. Portanto, os docentes tomarão ciência e serão sensibilizados acerca deste norteamento, de modo que poderão somar esforços para que os processos formativos sejam planejados e desenvolvidos para que os futuros profissionais desempenhem seu trabalho como resposta às necessidades de saúde da população e em consonância com o que preconiza o SUS, bem como com criticidade, ética e humanização.

Ainda sobre os encontros pedagógicos prévios, muitos professores que atuam nas escolas de educação profissional não foram formados para este trabalho, conforme já mencionado. A inserção na docência se dá de maneira um tanto precoce e sem a formação específica e necessária^(13,15)^. Assim, o preparo pedagógico anterior ao exercício docente oportuniza ao professor ampliar seu conhecimento tendo em vista o desenvolvimento de uma prática docente mais qualificada e experta, o que reverbera diretamente no processo de ensino, contribuindo para uma aprendizagem significativa.

Estudo realizado no contexto da graduação em enfermagem apontou que a formação dos enfermeiros é predominantemente voltada à assistência, com limitada ênfase na preparação para a docência, o que contribui, sobretudo no início da carreira docente, para a adoção de práticas pedagógicas baseadas em métodos tradicionais^(16)^. Tal evidência reforça a necessidade de criação de espaços pedagógicos voltados ao aperfeiçoamento do professor, bem como da oferta institucional de cursos que promovam a incorporação de recursos pedagógicos e tecnológicos à prática de ensino, de modo a favorecer uma aprendizagem crítica e reflexiva por parte dos alunos.

Nesse contexto, a licenciatura em enfermagem assume papel fundamental, ao buscar formar professores para a educação profissional técnica de nível médio, articulando conteúdos que contemplam tanto a formação do enfermeiro generalista quanto do enfermeiro docente^(17)^. Contudo, sua oferta é bastante reduzida em comparação ao bacharelado. Levantamento realizado em agosto de 2019 indicou que a licenciatura corresponde a apenas 1% (12) dos 1.168 cursos de graduação em enfermagem no Brasil^(18)^. Essa realidade evidencia a persistência da atuação de bacharéis na docência, o que reforça a fragilidade do reconhecimento da docência como uma profissão que demanda formação específica, devido à sua singularidade e complexidade próprias.

Nesse sentido de formação docente, as ETSUS também desenvolvem encontros pedagógicos no decorrer da atuação do professor, com a finalidade de acompanhar e verificar o progresso das aulas e orientar o docente em como melhor trabalhar determinado conteúdo, conforme manifestação e necessidade. O acompanhamento pedagógico permite ao professor a reflexão sobre a sua própria prática, o que promove aprendizado e amadurecimento, configurando-se, assim, como uma estratégia para o fortalecimento e a qualificação da atuação docente^(15)^. Nessa perspectiva, é possível ao professor buscar novos conhecimentos e estratégias de ensino-aprendizagem, aprimorando-os com criatividade e criticidade, e reorientando suas práticas educativas.

É oportuno ressaltar, mais uma vez, o quanto é indispensável e premente, para o exercício do magistério, a busca de conhecimentos que excedam os limites da profundidade de entendimento de uma matéria em particular. Ao apontar as sete categorias do Conhecimento Base para o Ensino, Shulman^(4)^ assinala saberes que são alicerçadores para a própria compreensão do professor, ou seja, para que ele entenda o que deve ser aprendido e como deve ser ensinado. Essa compreensão é elemento-chave para o aprendizado do estudante. Em outras palavras, a formação e a atuação do professor repercutem na formação do alunado.

Especificamente quando se trata da formação técnica, a busca de saberes que transpõem a enfermagem/saúde é basilar para que os docentes de enfermagem tenham uma atuação mais qualificada e que contribua para a melhoria da formação profissional e fortalecimento da enfermagem^(19)^. Ainda, é também impreterível, devido ao quantitativo expressivo e à relevância das atribuições dos trabalhadores técnicos no cuidado à saúde^(7,20)^. Logo, o ensino, sobretudo o de enfermagem, precisa ser encarado com a responsabilidade e a complexidade que a profissão reivindica, pois possui especificidades que demandam formação para o seu exercício.

Por fim, e ainda neste cenário da formação docente, as ETSUS desenvolvem encontros pedagógicos ao final da atuação do professor, após finalização dos módulos do curso ou ano letivo, para avaliação do seu trabalho. A possibilidade de refletir sobre as suas ações oportuniza ao docente uma análise de si e do seu exercício profissional na perspectiva de (trans)formação^(8)^. O processo formativo contínuo do docente, portanto, guarda relação com *feedbacks* da sua atuação enquanto professor, configurando-se como instrumentos potentes para autorreflexão e busca de conhecimento e qualificação na direção de uma prática docente mais experimentada.

Nesses encontros, os professores também têm a oportunidade de compartilhar demandas da sua prática, as quais são direcionadas, na maioria das vezes, a metodologias de ensino e avaliação. Aqui fica evidente que, para a docência, são necessários conhecimentos que vão além do conteúdo. Em sustentação, Shulman^(4)^ traz que o conhecimento pedagógico geral é um dos saberes constitutivos da base de conhecimentos para ensinar. Esse conhecimento permite ao professor acessar mais facilmente e eleger de forma mais adequada as metodologias de ensino, bem como selecionar apropriadamente os instrumentos para avaliação da aprendizagem^(2)^. Portanto, do professor é requerida formação contínua e permanente que lhe permita alcançar a qualificação necessária para o ensino.

No tocante ao desenvolvimento dos encontros pedagógicos, os coordenadores mencionaram alguns fatores dificultadores, como o quadro provisório de docentes e a ausência de uma ideia de rede fortalecida.

Referente ao quadro provisório de docentes, tendo em vista que a contratação é por tempo determinado, a dificuldade reside na descontinuidade da formação pedagógica dos professores. A vinculação docente às escolas de educação profissional, comumente, ocorre temporariamente, o que origina grande rotatividade de professores^(12)^. Por certo, tal rodízio compromete tanto a continuidade do ensino quanto o aprimoramento dos processos formativos.

Em estudo realizado no Rio Grande do Sul, detectou-se que a falta de vínculos seguros na carreira do docente da educação profissional é uma realidade, e cerca de 64% a 89,4% dos docentes possuem mais de um vínculo, repercutindo negativamente na prática pedagógica, que é uma atividade complexa que demanda tempo de preparo cotidiano, o que extrapola a carga horária do professor^(21)^.

Os coordenadores, ao apontarem esse fator de dificuldade que se relaciona com o tipo de contratação docente, chamam atenção para a necessidade de revisão dos tipos de vínculos contratuais estabelecidos pelas escolas de educação profissional. Tal revisão se faz essencial diante da instabilidade gerada por contratos temporários, que dificultam a permanência e o desenvolvimento contínuo dos professores no ambiente educacional. Soma-se a isso a precariedade dessas relações contratuais, frequentemente marcadas por baixos salários e inexistência de plano de carreira estruturado. Essa realidade compromete não apenas o engajamento e a valorização dos docentes, mas também a qualidade do ensino, uma vez que impede a construção de vínculos duradouros com a instituição e com os estudantes.

Em referência à ausência de uma ideia de rede fortalecida, esse fator dificultador, na visão do coordenador, acarreta falta de uma identidade pedagógica comum às ETSUS e insegurança em seus processos de trabalho. Portanto, sinaliza-se para a necessidade de as escolas trilharem caminhos próximos, a partir de um direcionamento mais uniforme na condução dos seus processos pedagógicos.

Sabe-se que as ETSUS estão organizadas em rede, a Rede de Escolas Técnicas do SUS (RET-SUS), instituída nos anos 2000, cujo dispositivo legal foi atualizado em 2009, sob a Portaria nº 2.970. Atualmente, a rede é constituída por 41 escolas, presentes em todos os estados brasileiros. Entre outros objetivos, visa integrar as ETSUS por meio do compartilhamento de conhecimentos e informações de interesse comum às escolas^(22)^. Nessa perspectiva, percebe-se que a RET-SUS dá e ao mesmo tempo fomenta um sentido de unidade e integração entre as escolas, de maneira que, por mais que estejam geograficamente distantes, estejam alinhadas.

Contudo, conforme dificuldade mencionada, o sentido e o posicionamento da unidade, bem como a integração proporcionada pela rede, precisam ser fortalecidos, a fim de que as escolas possam refletir uma convergência em seus processos educativos, com destaque àqueles relacionados à formação docente. Assim, terão um perfil pedagógico símil e convergente, o qual dará segurança aos gestores e coordenadores na condução do seu trabalho.

### Limitações do estudo

É oportuno mencionar que as limitações do estudo se referem à coleta de dados realizada exclusivamente com os coordenadores, à não verificação dos planos de aula em relação à metodologia de ensino proposta pelas ETSUS, bem como à ausência de análise das avaliações realizadas por docentes e discentes. Recomenda-se, portanto, que futuras pesquisas sejam conduzidas com o objetivo de ampliar a compreensão do objeto/tema, incorporando diferentes perspectivas e fontes de dados, a fim de fortalecer a validade e abrangência dos resultados.

### Contribuições para a área da enfermagem

O estudo coloca em evidência a necessidade de reconhecimento e melhoria da profissionalização do ensino, apontando, especialmente, para a importância e indispensabilidade da formação docente para a docência na educação técnica em enfermagem, sobretudo no contexto das ETSUS. A responsabilidade, a complexidade e os desafios de ensinar colocam a formação do professor como uma demanda cogente e contínua, tendo em vista a qualidade da formação profissional e do cuidado em enfermagem.

## CONSIDERAÇÕES FINAIS

Os espaços pedagógicos constituídos nas ETSUS para formação docente se concentraram em encontros pedagógicos que acontecem antes do início da atuação docente, no decorrer e ao final da mesma. Para o desenvolvimento desta formação, foram apontados fatores dificultadores, como o quadro docente provisório e ausência de uma ideia de rede fortalecida.

A qualificação para a docência é uma necessidade real no entendimento de que ensino é profissão que requer conhecimento e formação específicos. No cenário da formação técnica em enfermagem, tal necessidade ganha mais evidência, pois o professor está diretamente envolvido no percurso formativo desse trabalhador, que é essencial para o cuidado no contexto do SUS.

As ETSUS, por meio dos coordenadores pedagógicos, ao planejarem e implementarem encontros pedagógicos com a finalidade de promover a qualificação docente, assumem o quanto é necessário e importante desenvolver estratégias no cotidiano escolar que viabilizem a troca de conhecimento entre os professores, de maneira que possam exercer seu trabalho de modo mais profissional e experto. Vale destacar o quanto se faz imprescindível o envolvimento e compromisso dos docentes com sua própria formação, seja na participação de qualificações institucionais ou na busca pessoal por aperfeiçoamento.

Obviamente, os desafios existem, e sua superação é elemento decisivo na direção de formar professores qualificados que contribuam com a formação de futuros profissionais comprometidos com o fazer saúde no e para o SUS. Portanto, fica a referência de pensar e implantar ações mais convergentes, que se articulem entre si, em rede visando às possibilidades formativas e ao enfretamento dos desafios que ora se apresentam.

## Data Availability

Os dados de pesquisa estão disponíveis no corpo do artigo.
